# Editorial: Neuroinflammation and neurodegenerative diseases

**DOI:** 10.3389/fnins.2025.1561636

**Published:** 2025-02-14

**Authors:** Pradeep Kumar, Kiran Bhaskar

**Affiliations:** ^1^Clinical Research Unit, All India Institute of Medical Sciences, New Delhi, India; ^2^Department of Molecular Genetics & Microbiology and Neurology, University of New Mexico, Albuquerque, NM, United States

**Keywords:** neuroinflammation, neurodegenerative disease, biomarkers, neuroprotection, therapeutic interventions

Neurodegenerative diseases, characterized by the progressive loss of neuronal structure and function, are among the most devastating health challenges of the modern era (Gadhave et al., 2024). Disorders such as Alzheimer's disease (AD), Parkinson's disease (PD), multiple sclerosis (MS), and amyotrophic lateral sclerosis (ALS) share few common pathological hallmarks: neurodegeneration, neuroinflammation, and impaired brain integrity/connectivity (Cova et al., 2017). This complex interplay of immune-mediated responses in the central nervous system (CNS) integrity has emerged as a pivotal contributor to neuronal damage and disease progression (Jellinger, 2010). The growing body of research on this topic underscores the importance of unraveling the mechanisms of neuroinflammation and restoring brain homeostasis, which will pave the way for innovative therapeutic strategies.

This Research Topic, titled *Neuroinflammation and neurodegenerative diseases*, comprises 14 insightful contributions from leading researchers. Collectively, these studies explore the molecular and cellular underpinnings of neuroinflammation, the diagnostic potential of biomarkers, and promising therapeutic avenues. Additional insights are provided on how peripherally-derived risk factors [such as type 2 diabetes mellitus (T2DM), osteoarthritis, and Coronavirus Disease 2019 (COVID-19)] can have an impact on brain integrity/neuroinflammation. This editorial highlights the key themes and findings presented in this Research Topic.

## Unraveling molecular mechanisms

Among variety of neurodegenerative phenotypes, understanding the molecular basis of neuroinflammation is crucial for identifying therapeutic targets. Several studies in this Research Topic shed light on these mechanisms. For instance, the potential role of Calcitonin Gene Related Peptide (CGRP) in synucleinopathies offers insights into the molecular mediators of neuroinflammation (Alexoudi et al.). Likewise, the study on α*-Synuclein-mediated mitochondrial translocation of cofilin-1* elucidates how mitochondrial dysfunction and oxidative stress are intertwined in PD pathology (Yan et al.). The reciprocal roles of retro-elements in regulating memory and immunity, as discussed in one article, further deepen our understanding of the genetic and epigenetic interplay in neurodegeneration (Herbert). Such findings underscore the intricate molecular networks driving oxidative stress and neuroinflammatory processes.

## Inflammatory biomarkers and diagnostics

The pursuit of reliable biomarkers for early diagnosis and disease monitoring remains a cornerstone of neurodegenerative research. The diagnostic values of plasma cell-free nuclear DNA (cf-nDNA) and cell-free mitochondrial DNA (cf-mtDNA) for PD and multiple system atrophy, as explored in one study, highlight the promise of liquid biopsy approaches (Ying et al.). Another study associates higher serum lipoprotein-associated phospholipase A_2_ (Lp-PLA2) levels with cognitive impairment in PD patients, emphasizing the role of systemic inflammation markers in CNS diseases (Wu et al.).

Many peripheral causes can impact neuronal integrity and trigger neuroinflammation. For example, comprehensive magnetic resonance imaging (MRI) assessments revealing subtle brain findings in non-hospitalized post-COVID patients with cognitive impairment extend the discussion to post-viral neuroinflammatory syndromes (Fineschi et al.). Similarly, Ni et al. observed significant abnormalities in the connectivity and topology of large brain functional networks in T2DM patients. Liu et al. has observed a positive correlation between patients with osteoarthritis and Parkinson's disease. This underscores the relevance of neuroinflammation in broader contexts, including long-term sequelae of infectious, metabolic, and other peripheral degenerative diseases.

## Therapeutic approaches and interventions

Targeted therapies to mitigate neuroinflammation hold transformative potential for patients with neurodegenerative diseases. The neuroprotective effects of the aqueous extract of *Swietenia macrophylla* leaf in a PD murine model (Cardoso et al., 2024), along with the cognitive and mood-enhancing properties of diethyl butylmalonate in 5× familial Alzheimer's disease (FAD) mice, exemplify novel pharmacological interventions (Yuan et al.).

Non-pharmacological approaches also show promise. The regulation of microglia through physical exercise, as highlighted in one study, underscores the potential of lifestyle modifications as adjunctive therapies (Strohm and Majewska). These findings align with broader efforts to identify holistic and accessible therapeutic options.

## Neuroprotective strategies

Preserving neuronal integrity amidst the challenges of neuroinflammation is a primary objective in neurodegenerative research. The impact of sleep, anxiety, and depression on traumatic brain injury outcomes, analyzed in another contribution, offers insights into the broader psychosocial dimensions of neuroprotection (Fu et al.).

Additionally, the spatiotemporal consistency analysis of cerebral small vessel disease via resting-state functional MRI (rs-fMRI) provides a window into the vascular contributions to neurodegeneration, emphasizing the need for integrated neurovascular protective strategies (Yang et al.).

## Looking ahead

**Figure 1:**
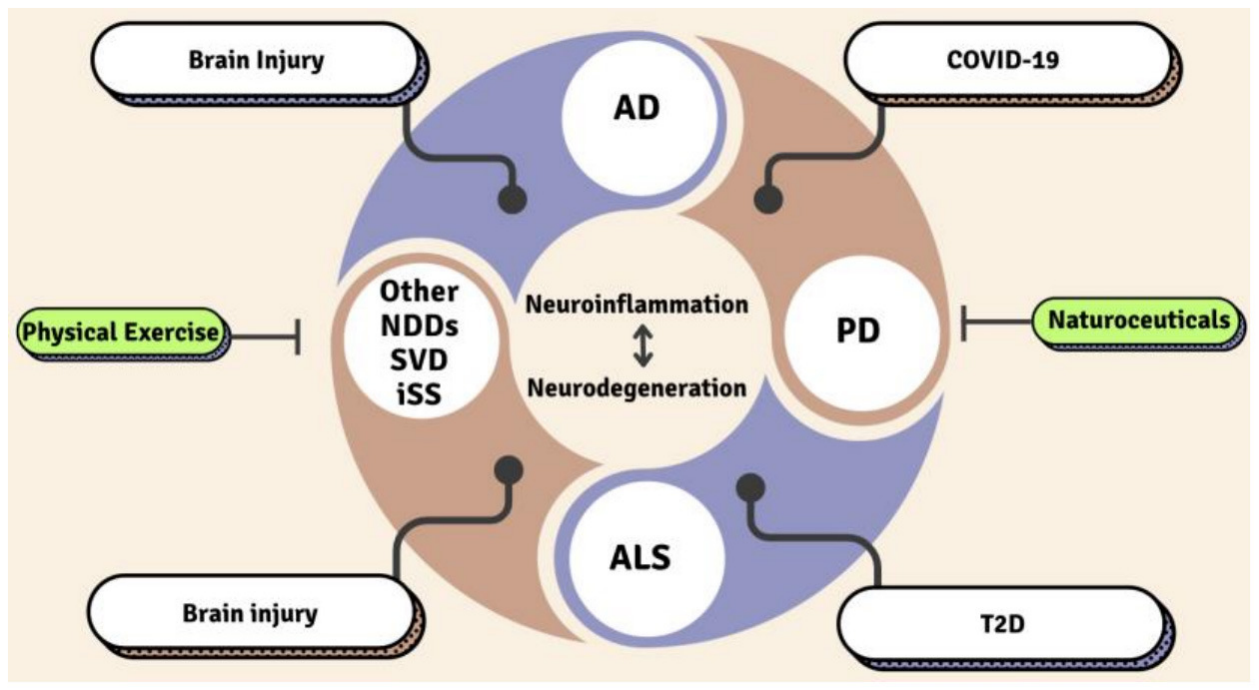
Regulators of neurodegeneration and neuroinflammation. SVD, Small Vessel Disease; AD, Alzheimer's Disease; PD, Parkinson's Disease; ALS, Amyotrophic Lateral Sclerosis; T2D, Type 2 Diabetes; iSS, Infratentorial superficial siderosis; NDDs, Neurodegenerative diseases.

This Research Topic represents a significant step forward in our understanding of neurodegenerative diseases with specific emphasis on neuroinflammation, metabolic condition, and peripheral diseases (Figure 1). The findings bridge gaps between basic science and translational research, providing a roadmap for future investigations. By identifying actionable targets and validating therapeutic interventions, these studies lay the groundwork for innovative solutions to one of modern medicine's most pressing challenges.

We extend our gratitude to all contributors for their invaluable insights and encourage continued interdisciplinary collaboration in this field. Together, we aspire to translate these scientific advances into tangible benefits for patients and their families, ultimately transforming the landscape of neurodegenerative disease management.
